# Medical Students and Personal Smartphones in the Clinical Environment: The Impact on Confidentiality of Personal Health Information and Professionalism

**DOI:** 10.2196/jmir.3138

**Published:** 2014-05-22

**Authors:** Kim Tran, Dante Morra, Vivian Lo, Sherman D Quan, Howard Abrams, Robert C Wu

**Affiliations:** ^1^University Health NetworkCentre for Innovation in Complex CareToronto, ONCanada; ^2^Trillium Health PartnersMississauga, ONCanada; ^3^Trillium Health PartnersInstitute for Better HealthMississauga, ONCanada; ^4^University Health NetworkDivision of General Internal MedicineToronto, ONCanada

**Keywords:** medical informatics, communication, hospitals, mobile phone, smartphones

## Abstract

**Background:**

Smartphones are becoming ubiquitous in health care settings. The increased adoption of mobile technology such as smartphones may be attributed to their use as a point-of-care information source and to perceived improvements in clinical communication and efficiency. However, little is known about medical students’ use of personal smartphones for clinical work.

**Objective:**

The intent of the study was to examine final-year medical students’ experience with and attitudes toward using personal mobile technology in the clinical environment, with respect to the perceived impact on patient confidentiality and provider professionalism.

**Methods:**

Cross-sectional surveys were completed by final-year medical students at the University of Toronto. Respondents were asked about the type of personal mobile phone they use, security features on their personal phone, experiences using their personal phone during clinical rotations, and attitudes about using their personal phone for clinical work purposes.

**Results:**

The overall response rate was 45.4% (99/218). Smartphone ownership was prevalent (98%, 97/99) with the majority (86%, 85/99) of participants using their personal phones for patient-related communication during clinical rotations. A total of 26% (26/99) of participants reported not having any type of security feature on their personal phone, 94% (90/96) of participants agreed that using their personal phone for clinical work makes them more efficient, and 86% (82/95) agreed that their personal phone allows them to provide better patient care. Although 68% (65/95) of participants believe that the use of personal phones for patient-related communication with colleagues poses a risk to the privacy and confidentiality of patient health information, 22% (21/96) of participants still use their personal phone to text or email identifiable patient data to colleagues.

**Conclusions:**

Our findings suggest that the use of personal smartphones for clinical work by medical students is prevalent. There is a need to more fully address the threat to patient confidentiality posed by the use of unsecured communication devices such as smartphones.

## Introduction

Smartphones are becoming ubiquitous in the health care setting. The rise in mobile technology such as smartphones may be attributed to perceived improvements in clinical communication, efficiency, and clinical skills [1-8]. Effective communication between health care providers is vital for optimal patient care. The importance of effective communication in the delivery of care is evident given that communication failures represent the most common cause of preventable disability or death [9].

Smartphones are also being recognized for their use in medical education and training. With smartphones being described as a “learn anywhere” resource [10], medical students and doctors are using medical-related applications for both educational and clinical purposes. Popular applications include those for medication/drug reference, disease diagnosis/management, and clinical scoring systems [11].

Although some studies have shown multiple benefits associated with increased connectivity from smartphone use, negative consequences of its use have also been described. Distracted doctoring due to frequent smartphone interruptions can result in adverse events such as medical errors [12,13]. Increasing use of personal smartphones for clinical communication has been observed, possibly due to the lack of an existing secure and efficient hospital communication system [3,14]. In addition, the use of personal smartphones for communicating patient information and the potential for unprofessional behavior have been described [3,15]. Finally, there are privacy concerns for patient health information to be communicated through unsecure methods such as email and text using personal smartphones [3].

This study explores the uses of personal smartphones by medical students during their clinical rotations and describes the perceived impact on the confidentiality of personal health information and professionalism.

## Methods

### Study Population

Participants were fourth-year medical students from the Faculty of Medicine at the University of Toronto. Participants would have been exposed to all of their clinical rotations in the various medical specialties.

### Survey Development

We developed the survey through an iterative process using standard survey methodology [16]. A literature search was conducted on MEDLINE to identify publications describing the uses of personal smartphones in the clinical environment (search terms: [cellular phone or smartphone or smart phone or iPhone or Android or BlackBerry or iPad or Windows mobile or personal digital assistant or mobile computer or mobile phone] AND [medical student or resident or physician] AND [medical education]). Semi-structured interviews were also conducted with seven medical students from the University of Toronto to examine their use of personal phones in the clinical environment. Important domains were identified and questions were generated through the literature review, interviews, and expert feedback. An expert group in the field of clinical informatics (RW, DM, VL, and SQ) reviewed the survey for content and face validity, comprehensiveness, and clarity. Pre-testing occurred with two focus groups consisting of individuals with research and/or design backgrounds who reviewed the survey for clarity and interpretation of individual questions. We then pilot-tested the survey with nine medical students and obtained feedback. The final survey consisted of 19 questions (Multimedia Appendix 1). A 5-point scale was used to express frequency for seven items and a 5-point Likert scale was used to express level of agreement for nine questions. The remaining three questions asked about the type, uses, and security features on medical students’ personal mobile phones.

### Data Collection and Analysis

In February 2013, medical students from the University of Toronto were surveyed during their final year of the medical school curriculum. Each student was provided with a paper survey at the beginning of his/her Transition to Residency course (all fourth-year medical students are required to take the course). A project manager for the Undergraduate Medical Education program distributed surveys at the beginning of class and completed surveys were collected during a class break. Students were informed that participation was voluntary and responses were anonymous and confidential. The study was approved by The University of Toronto Research Ethics Board. Descriptive statistics were generated from the survey results using Microsoft Excel.

## Results

### Uses of Personal Smartphones

The overall response rate was 45.4% (99/218). Nearly all (98%, 97/99) of the respondents currently owned a personal smartphone and the majority (79%, 78/99) of participants owned iPhones (Table 1).

Medical students reported using their personal smartphones for multiple purposes during their clinical rotations. The majority of students used their personal phone to communicate with medical team members about patient-related matters (86%, 85/99) and non−patient-related matters (93%, 92/99). Although 71% (70/99) of students had password protection on their phone, the survey revealed that 26% (26/99) of students’ phones lacked any type of security feature.

**Table 1:** The type, uses, and security features on medical students’ personal mobile phones (n=99).

Question	Answer options	n (%)
1. What type of personal mobile phone do you currently use?^a^	iPhone	78 (79)
	BlackBerry	6 (6)
	Windows Phone	0 (0)
	Android	14 (14)
	Cellular phone (non-smartphone)	2 (2)
	Other: Nokia smartphone	1 (1)
2. How do you use your personal mobile phone during clinical rotations?	Communication with patients	3 (3)
	Communication with other medical team members (patient-related)	85 (86)
	Communication with other medical team members (not patient-related)	92 (93)
	Medical references, resources, and applications	92 (93)
	View patient information	6 (6)
	Personal purposes (not work-related)	89 (90)
3. What type of security features do you have on your personal mobile phone?	Password protection	70 (71)
	Encryption	5 (5)
	I don’t know	6 (6)
	None	26 (26)

^a^Two participants reported using two types of personal mobile phones.

### The Disruptive Nature of Smartphones

A total of 46% (45/97) of medical students stated that they had answered/made a call, texted, or emailed on their personal phone during patient encounters (Table 2, Q1). However, 93% (89/96) of students perceived that their senior resident or attending physician interrupted patient meetings to answer/make a call, text, or email (Table 2, Q2). The disruptive nature of mobile phones also appeared to impact educational sessions with 31% (30/97) of medical students and 19% (18/96) of senior residents and attending physicians frequently interrupting an educational session to use their mobile phone (Table 2, Q3, Q4). In terms of personal use of their smartphones, 64% (61/95) of students frequently or always used their personal mobile phone for personal matters during their clinical rotations (Table 2, Q5).

**Table 2:** Participants’ experiences using personal mobile technology during clinical rotations (n=99)^a^.

Question	Never, n (%)	Rarely (1-3 times / month), n (%)	Occasionally (1-6 times / week), n (%)	Frequently (1-10 times / day), n (%)	Always (>10 times / day), n (%)
Q1. I have answered/made a call, texted, or emailed on my personal mobile phone while I was with a patient.	52 (54)	35 (36)	10 (10)	0 (0)	0 (0)
Q2. My senior resident or attending physician has interrupted a patient meeting to answer/make a call, text, or email.	7 (7)	38 (40)	41 (43)	10 (10)	0 (0)
Q3. I have answered/made a call, texted, or emailed on my personal mobile phone while I was in an educational session (eg, teaching rounds, bullet rounds, etc)	6 (6)	24 (25)	32 (33)	30 (31)	5 (5)
Q4. My senior resident or attending physician has interrupted an educational session to answer/make a call, text, or email.	3 (3)	41 (43)	34 (35)	18 (19)	0 (0)
Q5. I used my personal mobile phone for personal matters (eg, personal texts, calls, etc) during clinical rotations.	2 (2)	7 (7)	25 (26)	49 (52)	12 (13)
Q6. I used my personal mobile phone to text or email identifiable patient data (eg, patient last name, OHIP number, medical record number, etc) to colleagues.	75 (78)	17 (18)	3 (3)	1 (1)	0 (0)
Q7. My senior resident or attending physician has texted or emailed identifiable patient data to colleagues.	40 (44)	38 (42)	9 (10)	4 (4)	0 (0)

^a^A total of 99 surveys were returned but some participants did not answer every question.

### Communicating Patients’ Personal Health Information

In total, 78% (75/96) of students reported that they had never used their personal phone to text or email identifiable patient information to colleagues (Table 2, Q6). However, students reported that their senior residents or attending physicians were more likely to communicate identifiable patient information to colleagues, as only 44% (40/91) of students reported that their senior or attending had never texted or emailed identifiable patient information (Table 2, Q7). In terms of efficiency and patient care, 94% (90/96) of students believed that using their personal phone for clinical work made them more efficient and 86% (82/95) of students believed their personal phone allowed them to provide better patient care (Table 3, Q14, Q16). Although 68% (65/95) of students believed the use of personal phones for patient-related communication with colleagues poses a risk to the privacy and confidentiality of patient health information (Table 3, Q12), 22% (21/96) of participants still used their personal phone to text or email identifiable patient data to colleagues (Table 2, Q6). The majority of students (57%, 55/96) believed that the efficiency of communicating with colleagues through text and email using their personal phone outweighed the risk to the privacy and confidentiality of patient health information (Table 3, Q15).

**Table 3:** Participants’ attitudes about using personal mobile technologies for clinical work purposes (n=99)^a^.

Question	Strongly disagree, n (%)	Disagree, n (%)	Neutral, n (%)	Agree, n (%)	Strongly agree, n (%)
Q8. The medical school curriculum has educated me on appropriate and inappropriate ways to use my personal mobile phone for communicating patient information.	3 (3)	18 (19)	18 (19)	48 (51)	8 (8)
Q9. My senior resident or attending physician has given me feedback on appropriate and inappropriate ways to use my personal mobile phone for communicating patient information.	22 (23)	36 (37)	16 (17)	19 (20)	3 (3)
Q10. The medical school curriculum has educated me on appropriate and inappropriate ways to conduct myself professionally with mobile technology.	5 (5)	23 (24)	27 (28)	36 (38)	5 (5)
Q11. My senior resident or attending physician has given me feedback on appropriate and inappropriate ways to conduct myself professionally with mobile technology.	19 (20)	42 (44)	26 (27)	9 (9)	0 (0)
Q12. The use of personal mobile phones for patient-related communication with colleagues poses a risk to the privacy and confidentiality of patient health information.	2 (2)	7 (7)	21 (22)	38 (40)	27 (28)
Q13. My personal mobile phone is distracting during clinical work.	17 (18)	40 (42)	20 (21)	19 (20)	0 (0)
Q14. Using my personal mobile phone for clinical work makes me more efficient.	0 (0)	1 (1)	5 (5)	54 (56)	36 (38)
Q15. The efficiency of communicating with colleagues through text and email using my personal mobile phone outweighs the risk to the privacy and confidentiality of patient health information.	5 (5)	12 (13)	24 (25)	46 (48)	9 (9)
Q16. Using my personal mobile phone for clinical work allows me to provide better patient care.	0 (0)	0 (0)	13 (14)	59 (62)	23 (24)

^a^A total of 99 surveys were returned but some participants did not answer every question.

### Preparedness for Using Personal Smartphones in a Clinical Environment

A total of 59% (56/95) of students agreed or strongly agreed that their medical school curriculum had educated them on appropriate and inappropriate ways to use their personal mobile phone for communicating patient information (Table 3, Q8); 43% (41/96) of students believed their medical school curriculum had educated them on appropriate and inappropriate ways to conduct themselves professionally with mobile technology (Table 3, Q10).

## Discussion

### Principal Results

Personal smartphone use among medical students has become ubiquitous in health care settings. Our results provide a description of how and why medical students are using their personal smartphones. In addition, we describe the possible issues that could arise relating to medical students’ level of preparedness on the appropriate and inappropriate use of their smartphones in the clinical environment. Students are using their personal smartphones for work-related functions such as communicating with medical team members about patient-related and non−patient-related matters and using medical references, resources, and applications. They perceive that smartphone use increases their efficiency. While they communicate patient-related information using their personal phones, most medical students did not report communicating patient identifiable personal health information (PHI) in texts or emails. However, the majority (56%) of students reported that their senior residents and attending physicians had communicated patient identifiable PHI. In terms of preparedness, approximately half of students perceived they were educated on appropriate uses of their personal smartphones.

The personal smartphones of most participants lacked the necessary security features to protect the sensitive information that they may be sharing. As required by the Personal Health Information Protection Act (PHIPA), smartphones must be configured to operate in a secure manner when used to transmit or store personal health information [17]. Security features include the encryption of transmissions, password protection, and automated data wiping [17,18]. In recent years, the US Department of Health and Human Services has issued large fines to health care organizations and groups violating policies set out in the Health Insurance Portability and Accountability Act (HIPAA) [19]. These actions present a clear message that all health care providers and organizations will be held accountable for protecting their patients’ health information.

Despite security concerns over using personal smartphones for clinical work purposes, medical students perceive that their devices make them more efficient and allow them to provide better patient care. The majority believe that the benefits of perceived better care outweigh the possible harms of unsecure communication. However, this increased connectivity may have a negative impact on professionalism such as “distracted doctoring”, which may disrupt patient care and education.

Although a vast majority of medical students are using their personal smartphones in the clinical environment, many students do not feel that the medical school curriculum or role modeling has educated them on appropriate and inappropriate ways to use their personal smartphone for clinical work. There is increasing recognition that smartphone use by clinicians can be perceived to be unprofessional [2]. By answering their phone or responding to a text message during patient encounters, medical students and physicians can be perceived to be rude [20]. Through the medical school curriculum and role modeling, mobile etiquette should be taught to students so that they know where, when, and how it is appropriate to use their mobile technologies. Institutional policies regarding smartphone use in the clinical environment may also be beneficial [21]. This education would address issues of professionalism that can arise with the use of personal smartphones in clinical environments [12,13,15].

Our findings raise concerns over the security of personal health information. The use of personal smartphones for clinical work may increase efficiency, but there is concern about privacy breaches through unsecure sharing of confidential information. While individual clinicians including medical students, residents, and staff physicians need to understand the importance of keeping personal health information secure, it is the responsibility of the institutions to provide an effective, secure communication infrastructure for clinicians. Otherwise, we can expect ongoing privacy breaches.

### Limitations

This study has several limitations. The study only included medical students from a single university in Canada and our response rate was only 45.4%. We may also have a biased selection of medical students who own smartphones. However, the university is affiliated with five academic teaching hospitals. With a total of 99 responses, we believe that these results are likely to be generalizable to medical teaching institutions in Canada and the United States. The study also examined self-reported experiences. Participant responses were, therefore, subject to both recall bias and response bias. In addition, due to the sensitive nature of PHI, communication of PHI may be under-reported. Our findings show that students perceived their senior residents or attending physicians to communicate PHI more often than they reported for themselves. However, it is unclear whether the devices their senior residents or attending physicians were using to communicate PHI were personal phones or institutional devices.

### Comparison With Prior Work

Some of our findings are consistent with the literature reporting high use of smartphones by clinicians for work purposes. Consistent with previous literature, smartphones are being used for clinical and educational purposes and perceived improvements in efficiency have been reported [1-4,6]. Issues around interruptions resulting from smartphone use have also previously been described [22-24]. Additionally, professionalism issues have been described with medical trainees using smartphones in the clinical setting [20]. However, our study contributes original knowledge regarding *personal* smartphone use by medical students in the clinical environment: specifically, the prevalence of smartphone ownership among medical students, the various uses of personal smartphones, and students’ level of preparedness for using their personal smartphones in the clinical environment.

### Conclusions

The use of personal smartphones in the clinical environment is an established reality. It is evident that medical students prefer to use their personal smartphones for clinical work as they perceive that these devices make them more efficient and allow them to provide better patient care. With the popularity of personal smartphones, it is critical that more attention be focused on educating medical professionals on how to appropriately use their personal devices for clinical work as well as adopting secure means for clinical communication.

## Multimedia Appendix 1

Smartphone survey.

## Abbreviations

PHI: personal health information
