# Supplementary material for: Medical Students and Personal Smartphones in the Clinical Environment: The Impact on Confidentiality of Personal Health Information and Professionalism
Source: J Med Internet Res. 2014 May 22;16(5):e132. doi: 10.2196/jmir.3138 (PMC4051746; doi:10.2196/jmir.3138)
Supplement: Supplementary file 1 [file jmir_v16i5e132_app1.pdf]

Uses of Personal Mobile Technologies in a Clinical Environment

We are conducting a research study to measure your experiences and attitudes regarding the use of *personal* mobile technologies for clinical work purposes. We have received REB approval to conduct this study. Your participation is voluntary, you have the right not to participate and you can withdraw your participation at any time. If you decide not to participate, there will be no negative consequences. All responses are completely anonymous and will be aggregated. Your responses will remain confidential and will not be identifiable. Your responses will be kept indefinitely in digital format, and your paper survey will be destroyed five years after the study is complete.
Please note that by completing and submitting this survey, you are providing consent to participate in this study and for your survey results to be anonymously used for research publication and dissemination purposes.

If you have any questions about this study, please contact:

Kim Tran, Project Leader
Centre for Innovation in Complex Care, UHN
kim.tran2@uhn.on.ca

Section 1: Personal mobile technology

1. What type of personal mobile phone do you currently use? (check all that apply)
   - ☐ iPhone
   - ☐ Android
   - ☐ Blackberry
   - ☐ Cellular phone (non-smartphone)
   - ☐ Windows Phone
   - ☐ Other: ___________________________

2. How do you use your personal mobile phone during clinical rotations? (check all that apply)
   - ☐ Communication with patients
   - ☐ Communication with other medical team members (patient-related)
   - ☐ Communication with other medical team members (not patient-related)
   - ☐ Medical references, resources and applications
   - ☐ View patient information
   - ☐ Personal purposes (not work-related)
   - ☐ Other: ___________________________

3. What type of security features do you have on your personal mobile phone? (check all that apply)
   - ☐ Password protection
   - ☐ None
   - ☐ Encryption
   - ☐ Other: ___________________________
   - ☐ I don't know

Section 2: Experiences using personal mobile technology. These statements refer to the use of your personal mobile phone during clinical rotations. Please check one box per question.

	Statement	**Never**	**Rarely** 1-3x/mo	**Occasionally** 1-6x/wk	**Frequently** 1-10x/day	**Always** >10x/ day
4.	I have answered/made a call, texted or emailed on my personal mobile phone while I was with a patient.	☐	☐	☐	☐	☐
5.	My senior resident or attending physician has interrupted a patient meeting to answer/make a call, text or email.	☐	☐	☐	☐	☐
6.	I have answered/made a call, texted or emailed on my personal mobile phone while I was in an educational session (i.e. teaching rounds, bullet rounds, etc.)	☐	☐	☐	☐	☐

	Never	Rarely 1-3x/mo	Occasionally 1-6x/wk	Frequently 1-10x/day	Always >10x/ day
7. My senior resident or attending physician has interrupted an educational session to answer/make a call, text or email.	☐	☐	☐	☐	☐
8. I used my personal mobile phone for personal matters (i.e. personal texts, calls, etc.) during clinical rotations.	☐	☐	☐	☐	☐
9. I used my personal mobile phone to text or email **identifiable patient data** (i.e. patient last name, OHIP number, medical record number, etc.) to colleagues.	☐	☐	☐	☐	☐
10. My senior resident or attending physician has texted or emailed **identifiable patient data** to colleagues.	☐	☐	☐	☐	☐

Section 3: Attitudes about using personal mobile technology. These statements refer to your attitudes about using a personal mobile phone for clinical work purposes. Please check one box per question.

Statement	Strongly Disagree	Disagree	Neutral	Agree	Strongly Agree
11. The medical school curriculum has educated me on appropriate and inappropriate ways to use my personal mobile phone for communicating patient information.	☐	☐	☐	☐	☐
12. My senior resident or attending physician has given me feedback on appropriate and inappropriate ways to use my personal mobile phone for communicating patient information.	☐	☐	☐	☐	☐
13. The medical school curriculum has educated me on appropriate and inappropriate ways to conduct myself professionally with mobile technology.	☐	☐	☐	☐	☐
14. My senior resident or attending physician has given me feedback on appropriate and inappropriate ways to conduct myself professionally with mobile technology.	☐	☐	☐	☐	☐
15. The use of personal mobile phones for patient-related communication with colleagues poses a risk to the privacy and confidentiality of patient health information.	☐	☐	☐	☐	☐
16. My personal mobile phone is distracting during clinical work.	☐	☐	☐	☐	☐
17. Using my personal mobile phone for clinical work makes me more efficient.	☐	☐	☐	☐	☐
18. The efficiency of communicating with colleagues through text and email using my personal mobile phone outweighs the risk to the privacy and confidentiality of patient health information.	☐	☐	☐	☐	☐
19. Using my personal mobile phone for clinical work allows me to provide better patient care.	☐	☐	☐	☐	☐

Thank you for completing the questionnaire. Your participation is greatly appreciated.
